# Mild Hypophagia and Associated Changes in Feeding-Related Gene Expression and c-Fos Immunoreactivity in Adult Male Rats with Sodium Valproate-Induced Autism

**DOI:** 10.3390/genes13020259

**Published:** 2022-01-28

**Authors:** Tapasya Pal, Kathryn J. Laloli, Cushla A. Moscrip, Pawel K. Olszewski, Anica Klockars

**Affiliations:** 1 School of Science, University of Waikato, Hamilton 3240, New Zealand; paltapasya23@gmail.com (T.P.); kat.l@hotmail.co.nz (K.J.L.); cmos102@aucklanduni.ac.nz (C.A.M.); pawel@waikato.ac.nz (P.K.O.); 2 Centre for Neuroscience and Regenerative Medicine, University of Technology Sydney, Sydney 2006, Australia; 3 Department of Food Science and Nutrition, University of Minnesota, St. Paul, MN 55114, USA; 4 Department of Integrative Biology and Physiology, Medical School, University of Minnesota, Minneapolis, MN 55418, USA; 5 School of Health, University of Waikato, Hamilton 3240, New Zealand

**Keywords:** autism, feeding, food intake, oxytocin

## Abstract

A core yet understudied symptom of autism is aberrant eating behaviour, including extremely narrow food preferences. Autistic individuals often refuse to eat despite hunger unless preferred food is given. We hypothesised that, apart from aberrant preference, underfeeding stems from abnormal hunger processing. Utilising an adult male VPA rat, a model of autism, we examined intake of ‘bland’ chow in animals maintained on this diet continuously, eating this food after fasting and after both food and water deprivation. We assessed body weight in adulthood to determine whether lower feeding led to slower growth. Since food intake is highly regulated by brain processes, we looked into the activation (c-Fos immunoreactivity) of central sites controlling appetite in animals subjected to food deprivation vs. fed ad libitum. Expression of genes involved in food intake in the hypothalamus and brain stem, regions responsible for energy balance, was measured in deprived vs. sated animals. We performed our analyses on VPAs and age-matched healthy controls. We found that VPAs ate less of the ‘bland’ chow when fed ad libitum and after deprivation than controls did. Their body weight increased more slowly than that of controls when maintained on the ‘bland’ food. While hungry controls had lower c-Fos IR in key feeding-related areas than their ad libitum-fed counterparts, in hungry VPAs c-Fos was unchanged or elevated compared to the fed ones. The lack of changes in expression of feeding-related genes upon deprivation in VPAs was in contrast to several transcripts affected by fasting in healthy controls. We conclude that hunger processing is dysregulated in the VPA rat.

## 1. Introduction

Autism spectrum disorder (ASD) incorporates a cluster of symptoms, including impaired social interactions, communication difficulties, repetitive behaviours and intellectual disability. The umbrella term ‘spectrum’ includes a range of psychiatric and neurological (structural and functional) anomalies with varying severity. The underlying causes are diverse and encompass over 140 known genetic polymorphisms, immunological disorders and metabolic abnormalities, environmental factors and fetal chemical insults, all resulting in neurodevelopmental abnormalities with strikingly similar phenotypic symptomology.

Importantly, an increasing body of evidence suggests that dysregulation of neurotransmitters and neuropeptides plays an important role in neurodevelopmental abnormalities in ASD and contributes to behavioural impairments. Previous studies by Zieminska and others examined neurotransmitter changes in a rat model of valproic acid-induced autism and found that fetal VPA insult extensively disrupts early brain development and neurotransmitter balance [1,2,3,4].

One of the core and yet understudied symptoms of ASD is aberrant eating behaviour. This trait is so common that Leo Kanner, who coined the term ‘autism’, defined it as one of the characteristic ASD anomalies in the first paper describing the disorder [5]. Children with ASD have a five-fold greater risk of developing eating behavioural issues, with picky eating being most common [6]. The peculiar eating practices include dietary selectivity [7,8,9], rigidity, preference for specific foods and the manner in which they are served [7,10,11,12]. Altered sensory processing, social stimuli, food texture and composition, and presentation all affect the willingness of individuals with ASD to eat [10,12]. To add to the complexity of the problem, reward processing is altered in ASD. Lower density of the mesolimbic pathways has been described and—thus far—functionally linked with maladaptive responsiveness of the reward system to pleasant social cues in ASD [13]. In their fMRI study examining the neural basis of primary food-reward processing in ASD, Cascio and colleagues presented mildly hungry subjects with imagery of palatable food and found the anterior cingulate cortical (ACC) and insular activation to be higher in response to these cues [14]. ASD individuals receiving a monetary incentive upon correct responses to a given target paradigm display elevated activity in the left anterior cingulate gyrus [15].

Altered inflammatory responses have been observed in individuals with ASD [16,17] and this observation has been further corroborated in animal models of ASD [18,19,20,21], along with elevated oxidative stress responses. 

Valproic acid (VPA) at a therapeutic level has neuroprotective properties, as demonstrated by Chuang and colleagues in a series of studies in vitro [22] and in animal models [23,24,25]. However, prenatal exposure to VPA at a high dose can lead to an imbalance in redox status [26] and elevated pro-inflammatory cytokines [26].

Under pro-inflammatory conditions and/or under oxidative stress, the regulation of neurotransmitter and neuropeptide release may become altered and, in turn, may lead to aberrant changes in their modulation in response to environmental variation (in this case, food deprivation). One significant player in the regulation of inflammatory responses in the brain is IL6, which is reported to be elevated in the VPA animal model of ASD [27]. Interestingly, IL6 in the hypothalamus also regulates energy homeostasis and protects against obesity [28,29].

Feeding abnormalities have also been reported in the very scarce feeding-related studies utilising animal models of ASD. For example, Buchner et al. [30] found that the body weight consequences of a mutation of the axonal differentiation and guidance protein, Cntnap2, in mouse were greatly modified by the availability of a palatable diet. Fukuhara et al. reported that mice with a Mecp2-null mutation, a protein essential for normal neuronal functioning, exposed to a tasty diet exhibited extreme hyperphagia, associated with changes in dopamine system gene expression in the reward circuit [31]. Mice heterozygous for the postsynaptic neurotransmitter receptor targeting protein, Nbea, gene overconsumed sucrose and fat and were hyper-sensitive to a hypophagic action of naltrexone [32]. Finally, overconsumption of palatable sugar, saccharin and milk solutions occurred in rats whose autism was induced by in utero exposure to valproic acid (VPA) [33].

One of the most puzzling aspects of the extreme food selectivity in ASD is that it occurs despite leading to immediate and long-term problems, from gastrointestinal symptoms (including constipation, diarrhoea and abdominal discomfort) to severe micronutrient deficiencies and malnutrition. Importantly, this extreme selectivity—as reported by parents of children with the syndrome—involves refusal of acceptance of any other but the preferred food even after a period of energy deprivation, effectively becoming self-imposed food restriction. In fact, initial observation suggested that VPA ASD rats maintained on ‘bland’ chow eat amounts of this chow below what their healthy counterparts ingest [33].

This striking ability of ASD individuals to not eat despite fasting (unless preferred food is given) led us to hypothesise that, apart from aberrant reward mechanisms, this maladaptive behaviour is underpinned by abnormal hunger processing. Therefore, in the current set of studies utilising a common model of ASD, i.e., an adult male VPA rat, we investigated in detail the intake of ‘bland’ standard laboratory chow diet in animals maintained on this diet continuously, eating this food after a period of energy deprivation as well as after energy and water deprivation. We also assessed water intake, and we studied changes in body weight increases in adulthood to examine whether this lower food intake came with a cost of a slower growth trajectory. Since food intake is highly regulated by brain processes, we looked into activation (defined through c-Fos immunoreactivity) of key central sites responsible for feeding control in animals subjected to food deprivation vs. ad libitum-fed rats. Finally, we measured expression of select genes involved in food intake in the hypothalamus and brain stem (thus, two main regions responsible for energy intake) in deprived vs. sated animals. We performed our analyses in VPA animals as well as their age-matched healthy controls.

## 2. Materials and Methods

### 2.1. Animals

The experiments were conducted on Sprague-Dawley rats. The animals were housed in Plexiglas cages with wire tops in a temperature-controlled (22 °C) animal room with a 12:12 light:dark cycle (lights on at 07:00). Standard laboratory chow pellets (Sharpes, New Zealand; energy density: 3.6 kcal/g) and tap water were available ad libitum unless stated otherwise. Animals were treated in accordance with the National Institute of Health Guide for the Care and Use of Laboratory Animals. The University of Waikato Animal Ethics Committee approved the procedures described herein (Protocol# 1088).

### 2.2. Sodium Valproate Exposure

A well-established procedure (including the choice of VPA dose) was followed to generate VPA offspring [34,35,36,37]. Adult female Sprague-Dawley rats were mated overnight with age-matched Sprague-Dawley males (approximately 5–6 months old). Vaginal smears were stained with 1% crystal violet to detect spermatozoa and upon identification of spermatozoa, the date was designated as E0.5. Females received a single intraperitoneal (i.p.) injection of either 500 mg/kg sodium valproate (Sigma) or isovolumetric physiological saline (0.9% NaCl) i.p. on E12.5. Female rats treated with sodium valproate were healthy—similar to previous reports—and no significant difference between the litter sizes of sodium valproate-treated animals and those of controls was found. Females were allowed to nurse and raise their offspring until weaning on postnatal day (PND) 25. Sodium valproate-exposed offspring (from here on referred to as VPA animals) had no major morphological anomaly. However, in line with previous reports on VPA rats, approximately 12% of males developed crooked tails showing mild neural tube defects induced by prenatal VPA exposure [38]. Some VPA animals developed transient chromodacryorrhea, which was of no major concern [35]. Because autism is more pronounced in males [39,40], we used only males in the experiments.

### 2.3. Confirmation of ASD-like Traits in VPA Rats

In order to ensure that the prenatal VPA treatment indeed produced an ASD-like phenotype in the offspring, we performed select behavioural tests to identify relevant traits consistent with earlier reports on the VPA model of ASD. These tests were selected from the standard battery of behavioural approaches to identify autistic traits in VPA animals (for reference, see [34,41,42]). The tests used in our strategy to confirm the ASD phenotype included open field tests for social interactions and the elevated plus maze test for assessment of anxiety-like behaviour. The collective outcome of these ASD trait analyses confirmed that our VPA cohort indeed exhibited a set of abnormal behaviours in concert with the predicted phenotype.

#### 2.3.1. Open Field Test for Social Interactions 

A modified version of the behavioural test described previously by [34] was used, in which a control and a VPA rat (PND 35) were placed in an open arena of 44 × 44 cm dimensions for nine minutes and all sessions were recorded. Social interactions such as sniffing, licking, crawling over or under, mounting, anogenital inspections and approaching or following a healthy conspecific were scored for every animal participating in the experiments. 

#### 2.3.2. Elevated-Plus Maze Test 

In order to assess exploration of a novel environment, a modified version of a previously published protocol was followed [40], in which each rat (PND 30–35) was initially placed into the open field arena for 5 min before being placed in the elevated plus maze for another 5 min. The maze was elevated 50 cm above the ground and had two open arms and two closed arms of 50 × 10 × 40 cm dimensions. The arms were opposite each other and there was an open roof arrangement. The rat was always placed at the centre of the plus maze facing an open arm. The maze was cleaned with 70% ethanol before and after each rat was placed inside. The time spent and number of entries into the open arm was noted, along with the time spent in the closed arms for every rat. An entry into the open arm was defined as both paws being on or beyond the boundary of the closed arms. To assess whether the observation was merely a collateral effect of anxiety owing to a novel environment, self-grooming time was also evaluated as a parameter of anxiety-like behaviour [41]. 

### 2.4. Feeding Studies 

#### 2.4.1. Ad Libitum Standard Chow and Water Intake in VPA Rats versus Healthy Controls

Animals (*n* = 19/group, PND86, male, BW: ctrl 415.8 ± 8.6 g and VPA 398.7 ± 8.4) were individually housed and were allowed unrestricted access to standard laboratory chow and tap water. Chow and water were changed daily. During the time of the change, the amount of consumed water and food in the previous 24 h was recorded (chow and water bottles were weighed and the amounts subtracted from the values recorded 24 h earlier). 

#### 2.4.2. Energy Deprivation-Induced Feeding

Individually housed animals (the same cohort as described in Section 2.4.1) were deprived of chow overnight (water was available at all times). On the next day at 10:00 AM, chow was returned to the cages and chow and water intakes were measured after two hours of re-feeding.

#### 2.4.3. Feeding after Overnight Food and Water Deprivation

As a follow up to Experiment 2.4.2. (after a period of one week without any treatment), we wished to investigate whether concurrent deprivation of food and water affected feeding or drinking in VPA rats. Thus, individually housed rats had no access to food or water at night. At 10:00 on the next day, both tap water and standard chow were returned to cages and consumption was measured after 2 h.

#### 2.4.4. Body Weight Trajectory

Animals (*n* = 19/group) were maintained from weaning until PND225 on an ad libitum standard chow and water diet and their body weights were assessed weekly throughout that period. 

### 2.5. Neuronal Activation in Hungry vs. Fed Animals

#### 2.5.1. Deprivation and Tissue Dissection 

In the feeding studies described above we found that while VPA and control rats drank similar amounts of water, VPAs ate less of the standard laboratory chow after deprivation. In this study, we therefore wished to investigate changes in neuronal activation in feeding-related brain sites between hungry and ad libitum-fed rats. We investigated c-Fos immunoreactivity (IR; a marker of neuronal activation) in healthy control rats and in the VPA cohort, either having been deprived of food overnight or having unrestricted access to chow during this time. 

An independent cohort of animals (*n* = 10/group) was deprived of chow for 16 h with ad libitum access to water (referred to as ‘deprived’). Animals with ad libitum access to chow and water were used as reference (referred to as ‘ad libitum’; *n* = 9–10/group). Animals were anaesthetised with single i.p. administration of 35% urethane dissolved in 0.9% saline. Toe-pinch and palpebral reflexes were checked and transcardial perfusion was performed with 50 mL of saline followed by 500 mL of 4% paraformaldehyde in phosphate buffered saline (pH 7.4). Brains were excised and post-fixed overnight in the same aldehyde-based fixative at 4 °C. 60 μm-thick coronal sections were cut with a vibratome (Leica, Germany) and processed as free-floating sections for immunostaining of the protein in question (c-Fos). Immunohistochemistry for simultaneous detection of oxytocin and c-Fos was also performed from the same set of sections. 

#### 2.5.2. Immunohistochemistry

Immunohistochemistry was performed following the protocol previously published with some modifications [42]. 

Sections were rinsed in 50 nM tris-buffered saline (TBS, pH 7.4–7.6), and then pre-treated for 10 min in 3% H_2_O_2_, 10% methanol (diluted in TBS) at room temperature. After rinsing in TBS, they were preincubated for 1 h at room temperature followed by incubation at 4 °C for 48 h in primary rabbit-anti-Fos antibody (diluted 1:1500; Synaptic Systems, Australia). Sections were washed in TBS and incubated for 1 h at room temperature in biotinylated-secondary goat-anti-rabbit antibody (1:400; Vector Laboratories). Following four washes in TBS, sections were incubated for 1 h with avidin–biotin peroxidase complex (1:800; Elite Kit, Vector Laboratories). The medium for all incubations was a solution of 0.25% gelatin and 0.5% Triton X-100 in TBS. The peroxidase in the tissues was visualised with 0.05% diaminobenzidine (DAB, Sigma), 0.01% H_2_O_2_ and 0.3% nickel sulfate (15–20-min incubation). Sections were washed four times in TBS to stop the reaction, mounted onto gelatin-coated slides, air-dried overnight, dehydrated in ascending concentrations of ethanol followed by xylene, and embedded in Entellan (Merck KGaA, Darmstadt, Germany). 

Bright field images of immunohistochemically stained brain sections were acquired using an OMAX digital microscope camera attached to a Nikon Eclipse 400 microscope. Images were not processed digitally before or after the analysis; however, care was taken that the brightness, contrast and resolution of photographs used in software-based Fos-positive nuclear profile counting (Nikon NIS Elements image software) were similar. The number of Fos-positive nuclei per 1 mm^2^ was counted bilaterally for each neuroanatomical region of interest using ImageJ (Fiji), with boundaries defined according to the Paxinos and Watson brain atlas. Since the analysis of c-Fos immunoreactivity was conducted per mm^2^ of each site, the actual comparison of the activation levels reflected the density rather than the raw number of Fos-positive neurons. Therefore, any potential morphological changes (not evaluated here as c-Fos immunohistochemistry is not an appropriate method for combination with morphological analyses) did not impact the overall values reported here.

Image acquisition process, resolution and aspect ratio were kept constant using the same setup as described above. The number of total oxytocin-positive cells, cells with co-localised oxytocin and c-Fos and c-Fos per mm^2^ were counted using ImageJ. The percentage of active oxytocin cells was calculated as well. 

The following areas were analysed (in parentheses, anterior–posterior ranges of bregma levels of sections used to analyse each site are shown): Nacc Core—nucleus accumbens core (1.28–0.96); Nacc Shell—nucleus accumbens shell (1.28–0.96); AP—area postrema (−13.92 to −14.16); ARC—arcuate nucleus (−2.16 to −2.52); BLA—basolateral amygdala (−2.64 to −2.92); CEA—central nucleus of the amygdala (−2.64 to −2.92); DMH—dorsomedial nucleus of the hypothalamus (−3.00 to −3.24); DMNV—dorsal motor nucleus of the vagus (−13.76 to −14.16); NTS—nucleus of the solitary tract (−13.76 to −14.16); PVN—paraventricular nucleus of the hypothalamus (−1.56 to −1.92); SON—supraoptic nucleus (−0.96 to −1.2); VMH—ventromedial nucleus (−3.00 to −3.24); VTA—ventral tegmental area (−6.72 to −6.84).

### 2.6. Gene Expression in Hungry vs. Fed Animals

#### 2.6.1. Deprivation and Dissection of Hypothalamus and Brainstem 

This study was designed to investigate expression of feeding-related genes in the hypothalamus and brain stem of VPA and control rats that were either hungry or had unrestricted access to food. Experimentally naive control and ASD animals were divided into two groups each. One group (*n* = 10–12/group) had ad libitum access to standard chow and water, whereas the other was deprived of food overnight prior to being sacrificed by decapitation (*n* = 9–12/group) at 10:00 the next morning. Hypothalami and brainstems were dissected and immersed in RNAlater (Ambion, Thermo Fisher Scientific, Auckland, New Zealand) for 2 h at room temperature and the samples were then frozen at −80 °C until further processing.

#### 2.6.2. rtPCR Protocol and Data Analysis

A standard protocol for sample preparation and rtPCR was used, following a protocol previously described [43]. Tissues kept in RNAlater were homogenised in 400 µL Trizol (Ambion), after which 80 µL chloroform was added and samples were centrifuged at room temperature for 10 min at 10,000× *g*. The clear phase containing the total RNA was isolated and precipitated using 0.5 mL isopropanol in an ice bath for 10 min followed by centrifugation at 4 °C for 20 min at 10,000× *g*. The aqueous phase was carefully removed, keeping the pellets intact. The pellets were washed in 300 µL ethanol by centrifuging at 4 °C for 10 min at 10,000× *g*. The supernatant was removed carefully and the pellets were air dried.

Pellets were dissolved in 8 µL DEPC water and 1 µL DNAse buffer (dNature). Samples were then incubated with 1 µL DNAse (dNature) per reaction at 37 °C for 30 min followed by inactivation of DNAse with ‘stop buffer’ (dNature), incubating at 67 °C for 10 min. Removal of DNA was confirmed via PCR using HOT FIREPol Blend Master Mix (dNature), followed with agarose gel electrophoresis. Concentrations of RNA were measured (µg/µL) with a nanodrop.

cDNA was synthesised from RNA samples with iScript Advanced cDNA synthesis kit (BioRad). Quantification and purity of cDNA was determined using a Qubit 4 fluorometer (Invitrogen) and nanodrop. Quantitative real-time PCR reactions were performed in duplicate. An amount of 4 µL of 25 ng/µL cDNA template was used per transcript along with 1 µL of forward and reverse primers (5 µM) specific to that transcript (Table 1), 10 µL iTaq Universal SYBR Green Supermix (BioRad) and 4 µL MilliQ water. Expression of housekeeping genes (Histone H3.3, TBP, Tubulin β3) was used to analyse normalization factors. MilliQ water was used as a template for negative controls for each transcript. NCBI-BLAST^®^ was used to check the specificity of the primer pairs in silico prior to setting up the reactions.

The amplification protocol was as follows: denaturation at 95 °C for 15 min, followed by 45 cycles of 15 s at 95 °C, 15 s at the primer-specific annealing temperature and 30 s at 72 °C. Thermal profiles of the amplified transcripts were visualised using melt peaks where Tm analysis of the negative value of the change in relative fluorescence units (RFU) over the change in temperature (°C) was plotted (−dRFU/dT) to determine specificity of the primers in accordance with a given transcript and primer dimer. 

### 2.7. Data Analysis

Data from the food intake experiments were analysed via GraphPad Prism using independent two-sample Student’s *t*-tests considering homoscedastic distribution, as outliers were excluded from analysis using Grubb’s test of extreme studentised deviate. Significance level was set at *p* ≤ 0.05. 

Body weight was measured to normalise the food intake. Body weight between groups was analysed using independent two-sample Student’s *t*-tests considering homoscedastic distribution. Area under the curve was calculated and an independent two-sample Student’s *t*-test was used to analyse the data.

Densities of Fos-positive nuclear profiles (per mm^2^) were averaged per individual, and then per group. Data between the two groups (control ad lib vs. control deprived and ASD ad lib vs. ASD deprived) in each cohort were compared using independent two-sample Student’s *t*-tests considering homoscedastic distribution. Values were considered significantly different for *p* ≤ 0.05. 

Analyses of rtPCR data utilised BioRad CFX Manager software (BioRad); rtPCR results were normalised with housekeeping genes and the ΔCq values were analysed with a Student’s *t*-test. Values were considered significantly different when *p* ≤ 0.05.

## 3. Results

**Figure 1:**
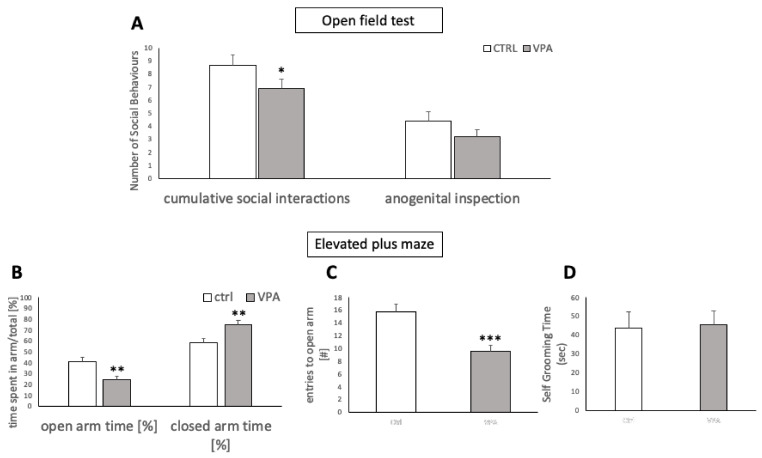
(**A**) Cumulative social interactions including sniffing, licking, crawling over or under, mounting, approaching or following the conspecific were assessed over a temporal window of nine minutes. ASD animals showed a significantly lower number of interactions. Anogenital inspections, evaluated separately, showed no significant difference. (**B**) Elevated Plus Maze. Time spent in the open arm, closed arm and (**C**) number of entries to the open arms were recorded. ASD rats spent a greater percentage of time in the closed arms and entered the open arms less often than controls. (**D**) No significant difference was detected in self-grooming duration. Data are expressed as mean ± SEM, *n* = 19/group. * *p* < 0.05, ** *p* < 0.01, *** *p* < 0.001.

Prior to undertaking the feeding studies, we conducted select behavioural tests in order to confirm that the VPA rats generated by the in utero pharmacological challenge indeed displayed a set of traits characteristic of autism. In those feeding-unrelated behavioural tests, VPA rats showed less interest in social interactions with conspecifics, which is in line with reduced sociality in autism spectrum disorder. The social interactions scored in our pre-test included sniffing, licking, crawling over or under, mounting, and approaching or following the conspecific, which showed a cumulative decrease in these socially driven behaviours (F = 1.411, df = 36, *p* = 0.0357). Separately evaluated anogenital inspections revealed no differences between VPA and control animals (Figure 1A). Subjected to the elevated plus maze (EPM), VPA animals spent a significantly larger amount of time in the closed arm (F = 1.53576, df = 36, *p* = 0.0013) with fewer entries into (F = 1.35718, df = 36, *p* < 0.0001), and less time spent in the open arm (F = 1.53576, df = 36, *p* = 0.0013), compared to controls (Figure 1B–D).

**Figure 2:**
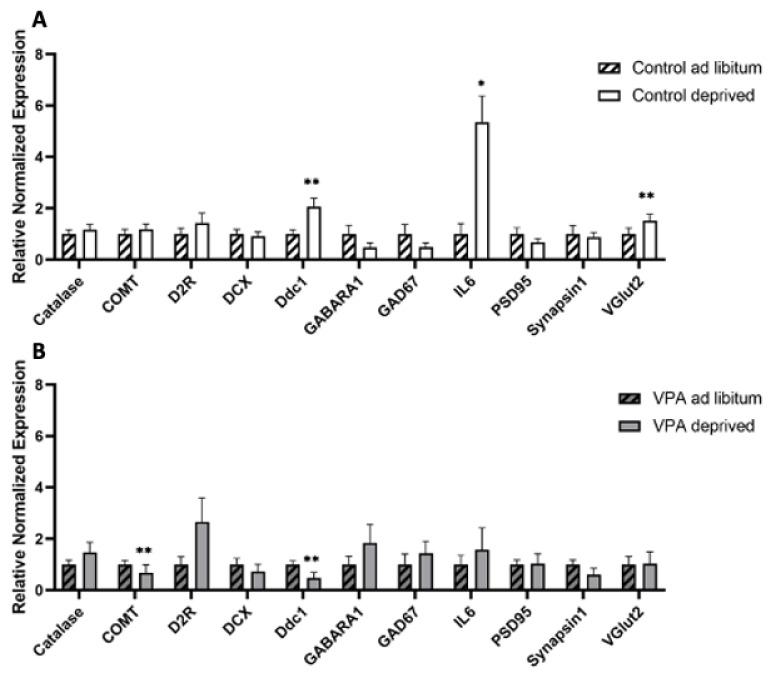
Gene expression changes in neurotransmitter receptors, oxidative stress and inflammatory markers analysed in (**A**) fed and unfed control animals and (**B**) fed and unfed VPA animals. COMT—Catechol-O-Methyltransferase, D2R—Dopamine Receptor D2, DCX—Doublecortin, Ddc1—DOPA decarboxylase, GABARA1—γ-aminobutyric acid type A receptor, GAD67—glutamate decarboxylase 1, IL6—Interleukin 6, PSD95—discs large MAGUK scaffold protein 4 (Dlg4), VGLUT2—vesicular glutamate transporter 2. Data are expressed as mean ± SEM, *n* = 9–10/group. * *p* < 0.05, ** *p* < 0.01.

rtPCR analysis of gene expression profiles showed that IL6 was upregulated (F = 5.162, df = 17, *p* = 0.0303) in hungry control animals compared to fed controls, while we did not see any change in the gene expression of IL6 in the VPA animals upon fasting. A similar pattern was observed with VGLUT2 expression (F = 1.451, df = 17, *p* = 0.0071), and Ddc1 expression was increased in hungry control rats (F = 1.09, df = 17, *p* = 0.0035), but decreased in VPA animals (F = 2.48115, df = 21, *p* = 0.0062) Finally, while there was no change in COMT expression between fed and hungry control animals, it was mildly down-regulated in unfed VPA animals compared to their fed counterparts (Figure 2). 

**Figure 3:**
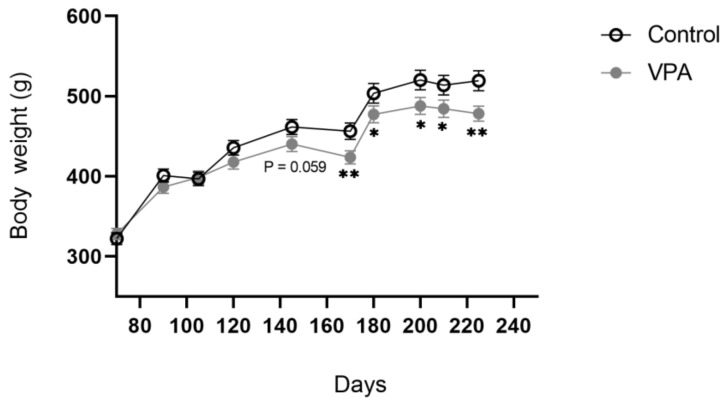
Body weight of adult ASD animals was significantly lower than that of healthy controls. Data are expressed as mean ± SEM, *n* = 19/group. * *p* < 0.05, ** *p* < 0.01.

Body weight analysis in adult VPA rats from PND 60 to PND 225 revealed a reduced area under the curve (AUC) (70,118.94 ± 858.85 for healthy animals versus 66,928.55 ± 803.65 in VPA animals; *p* = 0.015). A follow-up time point analysis showed differences in the rate of body weight increase in adult VPA rats compared to controls: while between PND60 and PND170 this did not reach significance, from PND170 onwards, VPA animals weighed less than controls (Figure 3). 

**Figure 4:**
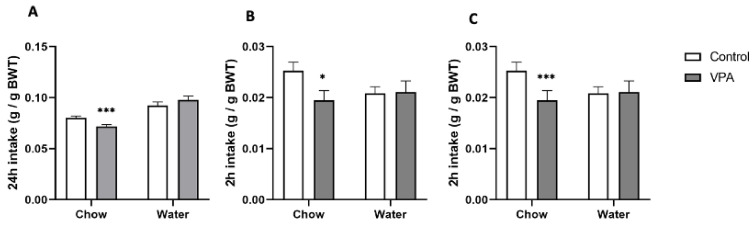
(**A**) 24-h chow intake of *ad libitum*-fed animals was significantly reduced in ASD rats. (**B**) Within 2 h of re-feeding after overnight chow deprivation, ASD animals exhibited significantly lower chow intake with no significant difference in water consumption. (**C**) Chow intake during 2 h of re-feeding after overnight chow and water deprivation was significantly reduced in VPA animals compared to healthy controls, while water intake was unaffected. Data are expressed as average intake ± SEM, *n* = 19/group. * *p* < 0.05, *p* < 0.01, *** *p* < 0.001.

Consistent with the body weight trajectory, ad libitum chow intake in VPA animals was significantly lower than in controls (F = 1.2, df = 36, *p* = 0.00087), as was deprivation-induced chow consumption regardless of whether only chow (F = 1.4, df = 36, *p* = 0.0145) or both chow and water (F = 1.48, df = 36, *p* = 3.71558 × 10^−5^) were withheld from the animals during the overnight fast. In all of these scenarios, water intake was similar between the VPAs and controls. It should be noted that chow intake experiments were conducted between PND68 and PND86; thus, during the timeframe when body weight differences were not yet significant (Figure 4).

**Figure 5:**
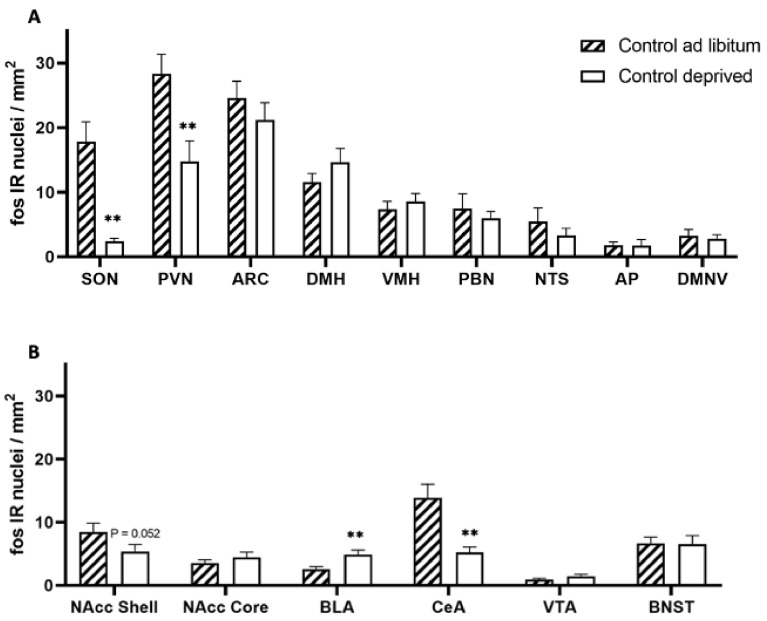
c-Fos immunoreactivity in ad libitum-fed and food-deprived healthy control animals. (**A**) PVN—paraventricular nucleus of the hypothalamus, SON—supraoptic nucleus, ARC—arcuate nucleus, LHA—lateral hypothalamic area, DMH—dorsomedial hypothalamus, VMH—ventromedial hypothalamus, PBN—parabrachial nucleus, NTS—nucleus of the solitary tract, AP—area postrema, DMNV—dorsal motor nucleus of the vagus, (**B**) Nacc Shell—Nucleus accumbens shell, Nacc Core—Nucleus accumbens core, BLA—basolateral amygdala, CeA—Central nucleus of the amygdala, VTA—ventral tegmental area, BNST—bed nucleus of the stria terminalis. Data are expressed as mean ± SEM, *n* = 9–10/group. *p* < 0.05, ** *p* < 0.01.

**Figure 6:**
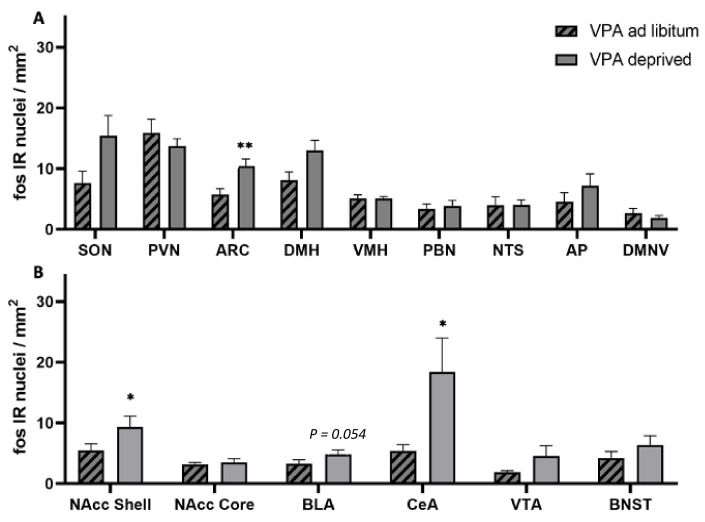
c-Fos immunoreactivity in ad libitum-fed and food deprived VPA animals. (**A**) PVN—paraventricular nucleus of the hypothalamus, SON—supraoptic nucleus, ARC—arcuate nucleus, LHA—lateral hypothalamic area, DMH—dorsomedial hypothalamus, VMH—ventromedial hypothalamus, PBN—parabrachial nucleus, NTS—nucleus of the solitary tract, AP—area postrema, DMNV—dorsal motor nucleus of the vagus, (**B**) Nacc Shell—Nucleus accumbens shell, Nacc Core—Nucleus accumbens core, BLA—basolateral amygdala, CeA—Central nucleus of the amygdala, VTA—ventral tegmental area, BNST—bed nucleus of the stria terminalis. Data are expressed as mean ± SEM, *n* = 9–10/group. * *p* < 0.05, ** *p* < 0.01.

**Figure 7:**
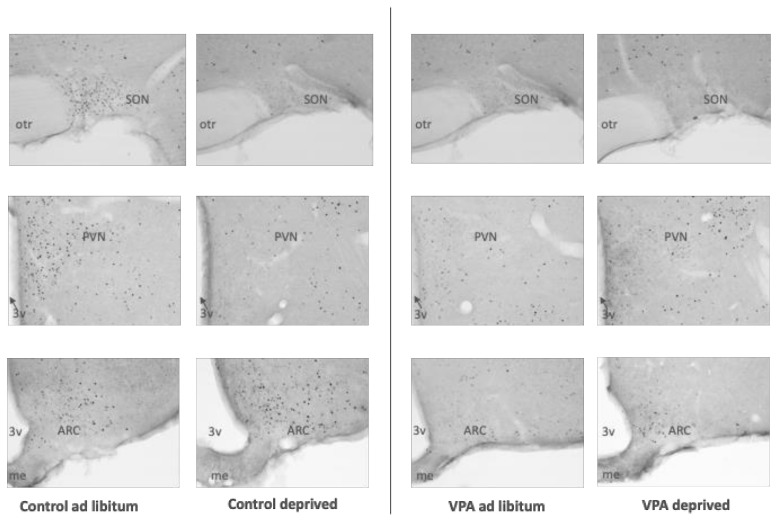
Photomicrographs depicting hypothalamic c-Fos IR. PVN—paraventricular nucleus of the hypothalamus, SON—supraoptic nucleus, ARC—arcuate nucleus, 3v—3rd ventricle, otr—optic tract.

**Figure 8:**
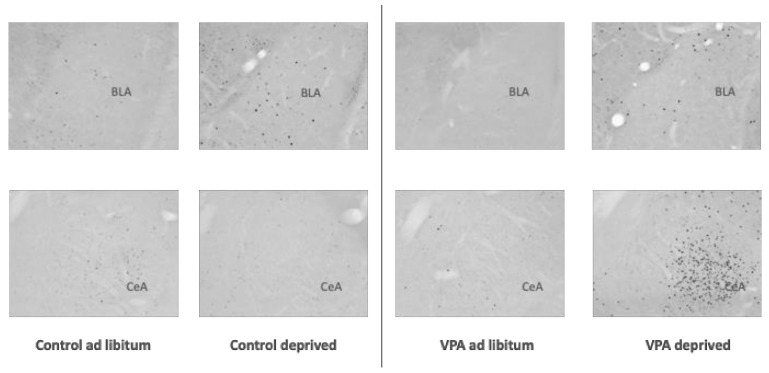
Photomicrographs depicting c-Fos IR in the amygdala. BLA—basolateral amygdala, CeA—Central nucleus of the amygdala.

**Figure 9:**
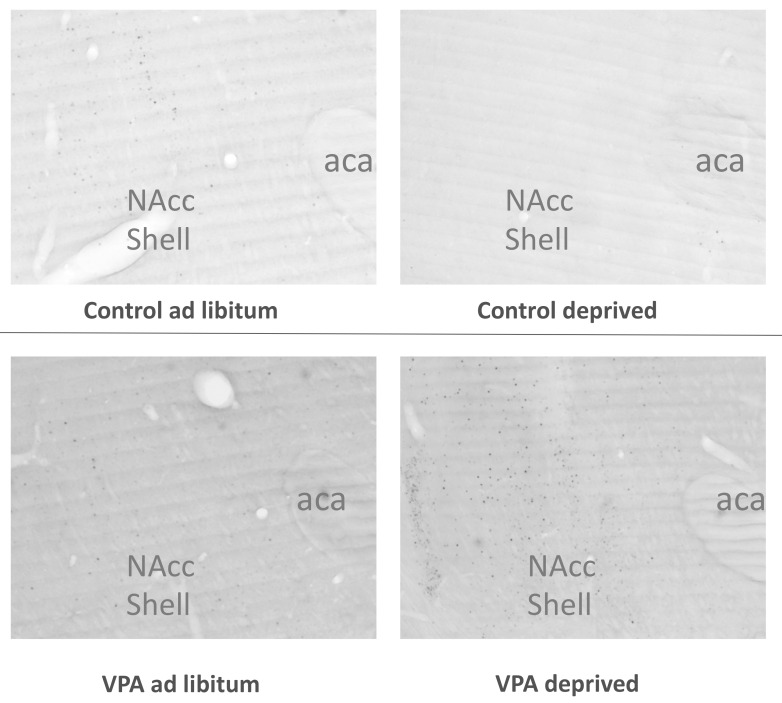
Photomicrographs depicting hypothalamic c-Fos IR in the Nacc Shell.

The analysis of c-Fos immunoreactivity (IR) in overnight-deprived versus ad libitum-fed rats in the healthy control cohort showed that compared to ad libitum-fed rats, the fasted animals had lower c-Fos IR levels in the PVN (F = 1.282, df = 15, *p* = 0.0038) and SON (F = 141.695, df = 15, *p* = 0.0085) as well as the CeA (F = 6.584, df = 14, *p* = 0.0011), and a strong trend towards reduced c-Fos IR in the Nacc Shell (F = 1.757, df = 15, *p* = 0.052). We also observed higher c-Fos IR in the BLA (F = 2.755, df = 16, *p* = 0.0051; Figure 5, Figure 6, Figure 7, Figure 8 and Figure 9). 

Surprisingly, unlike in healthy controls, in VPA fasted rats compared to ad libitum-fed VPAs, we did not see changes between the fed and deprived state in the PVN or SON. On the other hand, ARC c-Fos IR increased in deprived VPA rats (F = 1.317, df = 18, *p* = 0.0032). Furthermore, we noted an increase instead of a decrease in the Nacc Shell and CeA c-Fos IR (F = 2.453, df = 17, *p* = 0.035 and F = 30.994, df = 17, *p* = 0.022, respectively; Figure 6, Figure 7, Figure 8 and Figure 9).

RtPCR analysis of gene expression profiles in the hypothalamus and brain stem in healthy controls revealed that healthy food-deprived rats had upregulated hypothalamic AgRP and MOR expression compared to fed controls (F = 1.157, df = 17, *p* = 0.000287 and F = 1.498, df = 15, *p* = 0.000295, respectively), while hypothalamic OXT mRNA expression was decreased in deprived animals (F = 1.316, df = 15, *p* = 0.0397). Brainstem gene expression analysis revealed an increase in OXTR mRNA levels in unfed animals (F = 1.422, df = 15, *p* = 0.025). 

**Figure 10:**
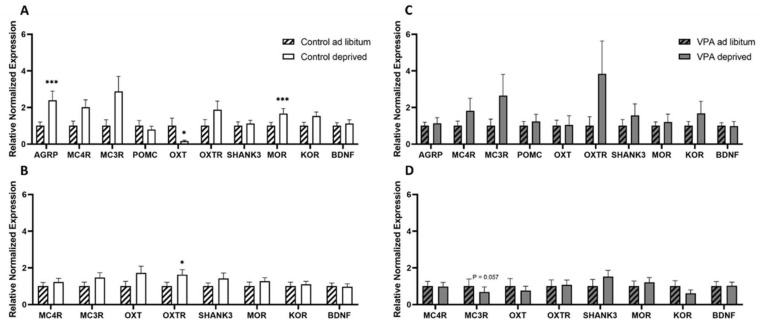
Differential gene expression patterns (**A**) in the hypothalamus of ad libitum-fed and food-deprived healthy control animals, (**B**) in the brainstem of ad libitum-fed and food-deprived healthy control animals, (**C**) in the hypothalamus of ad libitum-fed and food-deprived VPA animals and (**D**) in the brainstem of ad libitum-fed and food-deprived VPA animals. AgRP—agouti related protein, MC4R—melanocortin 4 receptor, MC3R—melanocortin 3 receptor, POMC—pro-opiomelanocortin, OXT—oxytocin, OXTR—oxytocin receptor, Shank3—SH3 And Multiple Ankyrin Repeat Domains 3, MOR—mu-opioid receptor, KOR—kappa-opioid receptor, BDNF—brain-derived neurotrophic factor. Data are expressed as mean ± SEM, *n* = 9–10/group. * *p* < 0.05, *p* < 0.01, *** *p* < 0.001.

Importantly, none of these genes’ expression levels differed between fed and unfed VPA animals and, instead, only brainstem MC3R expression in unfed VPA animals showed a trend toward statistical significance (F = 1.606, df = 18, *p* = 0.057; Figure 10).

## 4. Discussion

That the VPA rats responded with a less avid consummatory behaviour response when challenged with energy deprivation is of importance for understanding the pathophysiology of appetite dysregulation in ASD. This is because one of the greatest daily challenges for caregivers of individuals with ASD relates to providing a balanced diet that is accepted by a person under their assistance [44,45]. Acceptance of varied diets is very low in individuals with ASD [44,46,47,48]. To some extent, this is driven by sensory dysregulation. Indeed, high-functioning adults with ASD have been reported to avoid foods due to hypersensitivities to certain dietary item characteristics, such as taste, texture, smell or temperature [49]. Consequently, ASD individuals often receive a very limited diet consisting of a narrow range of their preferred foods. Intuitively, in the obesogenic environment in which highly palatable and caloric yet nutritionally substandard food is readily available, this often leads to overweightness or obesity. Interestingly, data show that many individuals with ASD are also underweight: a persistent avoidance of non-preferred food items in strategies that attempt to incorporate varied diets contributes to this phenomenon [50,51]. In fact, as shown in case reports, calorie self-restriction in ASD can at times be so severe that it requires medical intervention [52].

By using the VPA animal model of autism, Klockars et al. have recently reported that VPA rats overconsume sugar, saccharin and milk solutions [33], liquid diets that are highly preferred by rodents (see, e.g., [43,53]), whereas intake of a less palatable cornstarch solution is unaffected. The outcome of that study well reflected the phenomenon of reward hypersensitivity in humans. On the other hand, the current set of results showed that consumption of nutritious yet ‘bland’ standard laboratory chow was significantly lower in VPA ASD rats than in their healthy counterparts, thereby exemplifying reduced energy intake in autistic individuals upon presentation of non-preferred food. Importantly, this reduction was evident regardless of paradigm. In ad libitum-fed animals, chow consumption was approximately 15% lower than in controls. This reduction was chronic and persisted despite the fact that it translated into a reduced rate of body weight increase in these animals compared to their non-ASD conspecifics. Furthermore, VPA rats ate approximately 20% less than controls after an overnight period of energy deprivation (irrespective of whether water was or was not present during the period of having no access to chow). Thus, ASD animals consumed less both over the course of a meal when feeding was induced by a strong stimulus of energy deficit, and over 24 h periods of unrestricted access to chow when feeding was self-regulated. 

On the other hand, we did not see any difference in water intake between VPA and control rats. This was regardless of whether water consumption was measured in the ad libitum setting or in the meal scenarios prior to which water had been removed along with food or only food had been removed. Since drinking behaviour is typically affected by food consumption, one would expect water intake to be somewhat lower in VPAs as well. However, it should be noted that human studies have identified autism as a condition in which excessive water consumption occurs quite often in children and adults [54]. Thus, the lack of decline in water drinking in VPA rats, even though they were eating less food, was consistent with the findings in humans. 

Changes in the energy state of the organism (i.e., hunger versus satiety) are associated with changes in both brain activation and expression of genes, the latter especially within the hypothalamus and brain stem, the two regions that are key for the regulation of energy homeostasis. Here, we showed for the first time that VPA animals did not show a typical set of neuronal activation and gene expression patterns between the hungry and ad libitum-fed (sated) states.

Our gene expression analysis of inflammatory markers revealed that IL6 expression increased in healthy animals upon food deprivation, while we did not observe this change in VPA animals. Interestingly, IL6 has also been shown to improve glucose homeostasis and reduce body weight in obese animals [29,55], which points to its active involvement in food intake regulation [56] and may potentially amount to an orchestrated interplay of neuronal and glial cells in the context of neuroprotection [57], which is not observed in VPA animals and possibly feeds into the general pro-inflammatory environment of autistic brains. Furthermore, while no changes in COMT expression were observed between fed and hungry control animals, we found a decrease in the food-deprived VPA group. Importantly, Hersrud and Stoltenberg identified a correlation between COMT/dopamine dysregulation and maladaptive eating behaviour in undergraduate college women [58].

Earlier reports utilizing c-Fos immunohistochemistry showed that in hungry animals that are not anticipating a meal, the level of neuronal activation is significantly lower in many hypothalamic and extrahypothalamic sites, including the PVN and SON, than in refed or ad libitum-fed counterparts [59,60]. In the healthy control cohort of rats, we found that fasting indeed led to lower c-Fos expression in the PVN, SON, NAcc Shell (trend at *p* = 0.052) and CeA. The only site where c-Fos was elevated was the BLA, which possibly represented a higher motivational drive to search for food, other emotional aspects of fasting or the interplay between motivational and homeostatic feeding mechanisms [61,62]. On the other hand, in the VPA cohort, we did not see any hunger-associated decrease in any of the sites included in our study. In fact, we saw higher c-Fos IR in the ARC, CeA, BLA (trend at *p* = 0.054) and NAcc Shell, although—interestingly—average densities of Fos-positive nuclei in ad libitum-fed VPAs were similarly low to the densities in the hungry control cohort. While it remains to be elucidated whether the Fos pattern in ad libitum-fed VPAs reflected the overall lower food intake in these animals compared to controls or whether the lack of change in, e.g., hypothalamic activity underpinned VPAs’ weaker feeding response after deprivation, it is clear that the autistic animals’ feeding circuit was differently affected by the energy status of the animal. The notion of aberrant processing of the energy status in VPAs is further supported by the analysis of select feeding-related genes in the hypothalamus and brain stem. This is particularly relevant considering the fact that healthy controls showed significant changes in mRNA levels of genes associated with orexigenic (such as AgRP, mu-opioid receptor) and anorexigenic (oxytocin and oxytocin receptor) processes [63], whereas in VPA animals, only the melanocortin3 receptor transcript approached but did not reach (*p* = 0.057) significance.

One should note that the current paper focuses entirely on central processing of food intake and brain mechanisms that underpin hunger and satiety. However, ASD is characterised by abnormalities in not only the central nervous system, but also in—among others—the gastrointestinal system. In fact, ASD individuals are likely to have digestive issues, from constipation to diarrhoea [64,65,66], and the symptomology has been linked to both functional gut changes and microbiome alterations [65,67]. Thus, when considering brain data related to feeding, it should not be neglected that the brain integrates information derived from the gut directly (e.g., via vagal input) or indirectly (via endocrine communication). Therefore, the observed brain changes might not be the primary, but rather the secondary consequence of broader anomalies associated with ASD.

## 5. Conclusions

In sum, we conclude that adult male VPA animals ate less of the ‘bland’ chow when fed ad libitum as well as after deprivation. Their body weight increased more slowly than controls when maintained on this standard ‘bland’ food. While hungry control rats had lower c-Fos IR in key feeding-related areas than their ad libitum-fed counterparts, in hungry VPAs c-Fos was unchanged or elevated compared to the fed counterparts. The lack of changes in expression of feeding-related genes upon deprivation in VPAs was in contrast to several transcripts affected by fasting in healthy controls. This relative ‘unresponsiveness’ of the central circuits to changes in the energy status of the VPA organism may be an underlying factor in aberrant processing of hunger/satiation upon presentation of foods that do not belong to the preferred category.

## Figures and Tables

**Table 1:** List of all primers used in rtPCR experiments.

Housekeeping Genes		
Gene	Forward	Reverse
*TBP*	5′-AGAACAATCCAGATACAGCA-3′	5′-GGGAACTTCACATCACAGCTC-3′
*Tubulin β*	5′-TGCTGGCCATTCAGAGTAAGA-3′	5′-ACTCAGACACCAGGTCGTTCA-3′
*Actin b*	5’-AGTGTGACGTTGACATCCGT-3’	5’-TGCTAGGAGCCAGAGCAGTA-3’
Genes of interest		
Gene	Forward	Reverse
*AgRP*	5′- CAGAGTTCTCAGGTCTAAGTC-3′	5′-TTGAAGAAGCGGCAGTAGCAC-3′
*BDNF*	5′-TGCAGGGGCATAGCAAAAGG-3′	5′-CTTATGAATCGCCAGCCAATTCTC-3′
*Catalase*	5′-GGCTCTCACATAGCTGCCAA-3′	5′-TTACTGGTGAGGCTTGTGCC-3′
*COMT*	5′-TGTGTGCGGAACCTAAACGA-3′	5′-GAAGGTCGCGTGTTCCAGTA-3′
*D2R*	5′-ACCTGTCCTGGTACGATGATG-3′	5′-GCATGGCATAGTAGTTGTAGTGG-3′
*DCX*	5′-TCGTAGTTTTGATGCGTTGC-3′	5′-GCTTTCCCCTTCTTCCAGTT-3′
*Ddc1*	5′-ATGTGGCGTCATGTGTGTCT-3′	5′-CACGGCCACACAAAGAACAG-3′
*GABARA1*	5′-GGCTTGGGAGAGCGTGTAAC-3′	5′-CGTGACCCATCTTCTGCCAC-3′
*GAD67*	5′-GTGGCGTAGCCCATGGATG-3′	5′-ACTGGTGTGGGTGGTGGAAG-3′
*IL6*	5′- CTGCAAGAGACTTCCATCCAG-3′	5′- AGTGGTATAGACAGGTCTGTTGG-3′
*KOR*	5′-AGACCGCAACAACATCTACAT-3′	5′-GCACAGAACATCTCCAAAAGG-3′
*MC3R*	5’-TCCGATGCTGCCTAACCTCT-3’	5’-GGATGTTTTCCATCAGACTGACG-3’
*MC4R*	5’-CTTATGATGATCCCAACCCG-3’	5’-GTAGCTCCTTGCTTGCATCC-3’
*MOR*	5′-CGGACTCGGTAGGCTGTAAC-3′	5′-CCTGCCGCTCTTCTCTGG-3′
*OXT*	5′-GACGGTGGATCTCGGACTGAA-3′	5′-CGCCCCTAAAGGTATCATCACAAA-3′
*OXTR*	5′-GATCACGCTCGCCGTCTA-3′	5′-CCGTCTTGAGTCGCAGATTC-3′
*POMC*	5′-CAGGACCTCACCACGGAAAG-3′	5′-GTTCATCTCCGTTGCCTGGA-3′
*PSD95*	5′-CTTCTCAGCCATCGTAGAGG-3′	5′-GAGAGGTCTTCAATGACACG-3′
*Shank3*	5′-TACAGCACTTGGAGCACCTG-3′	5′-GTAATTGCGGACGTCCTTGT-3′
*Synapsin1*	5′-CACCAGGATGAAGACAAGCA-3′	5′-GTCGTTGTTGAGCAGGAGGT-3′
*VGlut2*	5′-CGTGAAGAATGGCAGTATGTCTTC-3′	5′-TGAGGCAAATAGTGCATAAAATATGACT-3′

## Data Availability

The data presented in this study are available on request from the corresponding author.
